# Community-Based Wound Care Programs for Unhoused Individuals

**DOI:** 10.1007/s44197-023-00157-6

**Published:** 2023-10-17

**Authors:** Taichi Goto, Christina Wang, Catherine Kwiat, Christopher Nguyen, Leorey N. Saligan

**Affiliations:** 1 National Institute of Nursing Research, National Institutes of Health, Bethesda, MD 20892 USA; 2 Hawaii Health & Harm Reduction Center, Honolulu, HI USA; 3 National Institute of Nursing Research, National Institutes of Health, 3 Center Drive, Building 3, Room 5E14, Bethesda, MD 20892 USA

**Keywords:** Wound care, Homelessness, Community-based care, Substance use, Street medicine

## Abstract

Wound care management for unhoused individuals is challenging due to the lack of healthcare infrastructure to handle the unique needs of this population. Therefore, we aimed to obtain insights for best practices and to establish a care clinic that is low threshold, community-based and meets the needs of unhoused people. We employed two approaches: (1) conduct a targeted narrative review of the literature of existing or proposed community-based program models that can address the wound care needs of unhoused individuals, and (2) assess cost-effectiveness and describe the results of a survey administered to unhoused clients and their health care providers at a community-based wound care program in Honolulu, Hawai'i. The literature search and screening yielded 11 articles relevant to the topic. Per the literature, existing community-based healthcare programs were successful when: (1) wound care services were incorporated into a broader social/health program, (2) cost-effective, and (3) comprehensive services were provided. Survey results in Honolulu found that the wound care program matched the needs of the targeted population and was cost-effective. Difficulty in following clients until wound closure and the sustainability of the program, particularly the lack of insurance reimbursement for street-based services, were perceived challenges. Additionally, the lack of insurance reimbursement for street-based wound care services continues to impact sustainability. Community-based programs can be successful in addressing the wound care needs of unhoused individuals if they address complex fundamental issues. This paper highlights existing gaps in logistics and policies that must be addressed to meet the specific medical needs of these vulnerable individuals.

## Introduction

In recent years, the increasing number of unhoused individuals in the USA has risen to become a pressing public health concern, capturing the attention of clinicians, researchers, and policymakers [1]. In 2020, 580,466 people in the USA were unhoused based on the Annual Homeless Assessment Report to the US Congress [2]. Due to foundational issues that this population faces (e.g., unstable housing, high risk for substance abuse, and behavioral health problems), unhoused individuals over-rely on emergency medical services, which can be costly and ineffective compared to outpatient and preventative care services [3–6].

The effects of homelessness on mental and physical health remain challenging to address [7, 8]. Chronic non-healing wounds are commonly observed in this population and are often associated with trauma, underlying intravenous drug abuse, chronic illnesses, malnutrition, residential instability, and/or unhygienic living conditions [3, 8–12]. Although some community-based primary and preventative healthcare services are available to address several of these health concerns, very few of these existing programs focus specifically on the wound care needs of this population [11, 13]. In addition, many unhoused individuals report negative health care experiences when accessing primary care due to social stigma [9, 14, 15]. Further, the administrative and structural complexity of health care systems can dissuade unhoused individuals from obtaining care for chronic wounds [16, 17]. These findings suggest that new perspectives, approaches, and interventions are needed to address the specific needs of the unhoused population.

Smaller, community-based wound care centers and clinics can be viable alternatives to the emergency department (ED) or hospital-based services by ensuring patient-provider trust through empathy and respect; broadening community outreach; providing culturally competent health education; and increasing the availability of drop-in services [9, 15]. To comprehensively explore current best and emerging practices in such programs, this paper employs a two-pronged approach: (1) conduct a narrative review of the literature of existing and proposed community-based program models that can address the wound care needs of unhoused individuals, and (2) evaluate the results of surveys administered to clients seen in a community-based wound care service for unhoused individuals and their health care providers in Honolulu, Hawai'i.

## Methods

### Literature Review

The search from PubMed, CINAHL Plus, and Scopus databases for this targeted narrative literature review included the following terms:

("wound care" AND "homelessness") OR ("syringe exchange program" AND "homelessness") OR ("soft tissue infection" AND "homelessness") OR ("wound care" AND "injection drug use") OR ("community based wound care" AND "injection drug use") OR ("soft tissue infection" AND "syringe exchange program") OR ("community-based care" AND "homelessness") OR ("care" AND "unhoused") OR (“wound” AND “syringe exchange program”)

to identify community-based programs that could be useful to establish wound care programs, particularly for unhoused individuals.

### Community-Based Wound Care Program in Honolulu, Hawai'i

#### Program Overview and Ethical Considerations

A couple of surveys were conducted by one of the authors (CW), who is the wound care nurse practitioner of a community-based wound care service that is integrated with the Hawai'i state syringe exchange program, formerly named the Community Health Outreach Work (CHOW). Since the administration of this survey, the CHOW Project has merged with the Life Foundation and is now known as Hawaii Health & Harm Reduction Center. One survey was administered to clients of the CHOW project who sought care during the intervention period from June 2016 to January 2017 consisting of two clinic days per week and three health fairs. A total of 116 clients were seen, with an average of at least two visits per client over the intervention period. The surveys were administered to evaluate the CHOW services. The target population of the first survey was intravenous drug users (IDUs) with wounds who accessed the CHOW services in downtown Honolulu. A second survey was administered to evaluate the concerns and needs of healthcare staff providing care across Honolulu to clients seen in the CHOW project. Both surveys were reviewed and deemed exempt by the institutional review board of a local university because the intent of the needs assessment surveys is to evaluate and improve procedures in providing free services to clients of the CHOW project.

#### Data Collection

*Needs assessments* The CHOW multidisciplinary team, including healthcare providers and social workers knowledgeable about wounds in clients seen in the syringe exchange program (SEP) population, developed a needs assessment survey for clients. The social worker administered and helped clients complete the needs assessment survey, which captured the self-reported frequency of wounds, type of wounds, and the number of times the client visited the emergency department or other clinics. The survey also asked about where the client receives wound care services, whether they would seek wound care through a CHOW community-based program, whether the client thinks they need help with wound care or needs wound supplies, and what some of the barriers are to accessing wound care services.

Additionally, a modified short form needs assessment was developed and administered to O’ahu wound care providers via email containing an online survey link. The provider needs assessment was used to assess the frequency of wounds seen, types of wounds, cause of wounds, barriers to providing wound care, whether he/she would support a community-based wound care program, and any additional recommendations.

*Client encounter data*. During the initial encounter at the CHOW clinic, the following information was collected by care providers, as part of the routine care intake: the client’s CHOW ID, current use of injectable drugs, wound characteristics including onset, location, and duration of wound(s), presence of wound pain; signs and symptoms of infection, prescribed antibiotics (if any), size of the wound, presence of wound undermining/ tunneling, and pertinent co-morbidity (e.g., diabetes). Additionally, other key information was also collected, including prior treatment facility, previous types of wound treatments (e.g., wound dressings), prior diagnosis and treatment plan, and prior medical referrals.

*Cost analysis*. Cost data were tracked through medical records accounting for the amount of funds spent for supplies and resources used for each client in CHOW. Extant data were used to estimate the cost of ED visits specifically for care afforded to IDU-related wounds. Additionally, extant data were obtained to assess ED utilization and related cost for opioid abuse/dependence and associated infections of wounds.

## Results

### Literature Review for Community-Based Programs

The literature search yielded a total of 74 articles. A review of the titles, abstracts, and full texts of these articles yielded 11 final articles that were relevant to this narrative review. Table 1 lists the community-based programs found in the targeted, narrative literature search that could be useful for wound care management, particularly for unhoused individuals. Common themes were evident from the reviewed articles. Existing community-based programs with or without wound care services were successful when: (1) wound care services were incorporated in a wider social or health program, (2) specific social or health programs with or without wound care services were cost-effective, and (3) comprehensive services were provided to address the complex foundational issues surrounding unhoused individuals.

**Table 1:** Community-based programs from the narrative literature search

Program name	Location	Services offered
Wound and Abscess Clinic at Casa Segura/Safehouse [30]	Oakland, California	Syringe exchange; soft tissue infection treatment (incision and drainage, abscess care, wound check, medical discussion, cellulitis treatment, aftercare, and chronic ulceration treatment); referrals to diagnostic testing, psychiatric or day treatment, parenting classes, and housing
Community-Based Medication-First (CBMF) Program [32]	This program was a concerted effort that took place across 6 existing community health sites in Washington State: Tacoma, Seattle, Centralia, Spokane, Walla Walla, and Kennewick	Drop-in, same-day visits; same-day starts for opioid user disorder medications; syringe exchange; HIV case management; primary care medical services; clothing and food pantry; and housing services
Baltimore Needle Exchange Program (BNEP) Wound Clinic [27]	Baltimore, Maryland	Wound treatment (wound assessment, wound cleaning, incision and drainage of acute abscesses, sharp debridement of chronic ulcers, compression treatment, antibiotic prescription, and specialized wound dressing); needle exchange; and hospital referrals for patients with severe chronic wounds
Dhaka Needle/Syringe Exchange Program (NSEP) [18, 50]	Dhaka, Bangladesh	Needle/syringe exchange; abscess management; HIV/AIDS education; rest and recreational facilities; and diagnosis and treatment of sexually transmitted diseases
Malmo Syringe Exchange Program (SEP) [20, 51]	Malmo, Sweden	Syringe exchange; education on overdose management; naloxone kit distribution; basic healthcare including gynecological care; social counseling; referrals to addiction treatment; and testing for sexually transmitted diseases and bloodborne infections
Infectious Disease Elimination Act (IDEA) Syringe Services Program (SSP) [33, 34]	Miami, Florida	Syringe exchange; distribution of safe injection kits, naloxone packs, and harm reduction packs; basic wound care; referrals to drug rehabilitation; and HIV and hepatitis C testing
Tacoma SEP [21]	Tacoma, Washington	Syringe exchange; distribution of naloxone packs, safe sex supplies, and first aid and hygiene kits; referrals for abstinence-based and medication-assisted treatment; assistance in learning about social and health services; and HIV and hepatitis C testing
Homelessness Team Program at Cohealth [23]	Melbourne, Australia	A wide range of community health services including podiatry, general practice, nursing, dental, physiotherapy, social work, occupational therapy, dietetics, diabetes education, and counseling
Health Care for the Homeless [35]	Baltimore, Maryland	Medical care; mental health and addiction counseling; case management; chronic disease management; occupational therapy; and assistance to secure housing
JustHealth Recuperative Care Program [26]	Seattle, Washington	Housing (a motel room was provided to all patients admitted to the program); nursing care; and case management
San Antonio Street Nursing [25]	San Antonio, Texas	Primary care medical services to unhoused populations

#### Wound Care Service Incorporated in Existing Programs

Five studies describing existing needle/syringe exchange programs have the potential to provide wound care services to unhoused individuals. Three programs, the Baltimore Needle Exchange Program (BNEP) Wound Clinic, the Dhaka Needle-Syringe Exchange Program (NSEP), and the Infectious Disease Elimination Act (IDEA) Syringe Services Program (SSP), offered wound services. Two programs, the Malmo Syringe Exchange Program (SEP) and the Tacoma SEP, did not mention wound care services, but the successes of these programs could easily translate to a successful wound care service, if added. A number of these needle/syringe exchange programs have shown positive results in their communities. The NSEP in Dhaka, Bangladesh, was part of several drop-in centers established in 1998 [18]. In addition to a needle/syringe exchange program, these centers offered treatment and education services for sexually transmitted infections as well as wound abscess management [18]. The Dhaka NSEP has contributed to reducing human immunodeficiency virus (HIV) prevalence among IDUs in Dhaka, compared to other countries in South Asia [18, 19]. A study describing the SEP in Malmo, Sweden, saw similar results, where the authors found that the SEP helped treat hepatitis C virus among IDUs [20]. Another study described the SEP in Tacoma, Washington, where the authors found that, despite substantial increases in factors associated with syringe sharing, such as depression and injection of amphetamines, rates of injection risk behavior remained stable across a four-year period, from 1997 to 2001 [21]. Further, though homelessness had become substantially worse in Tacoma by 2001, the rates of injection risk behavior remained stable, providing evidence that the Tacoma SEP was able to mitigate drug injection risk despite deteriorating social conditions [21]. Although the study did not focus specifically on wounds, the success of this SEP infers that mitigating injection risk behaviors may also aid in wound prevention, since practices such as sharing and reusing syringes increase the risk of wounds and infections [22].

#### Cost-Effectiveness of Community-Based Programs

The Homelessness Team Program at Cohealth in Melbourne, Australia, used active outreach to effectively treat foot wounds in the unhoused population through its no-cost podiatry service [23]. Since walking is often the main means of mobility for unhoused individuals, small podiatric issues can often compound into more severe wounds if left untreated. The Cohealth podiatry service sets up drop-in clinics at sites where unhoused people gather, so that they can receive primary healthcare for foot wounds and infections before they develop into more severe problems that require more invasive and expensive procedures [23]. This not only burdens the client but also increases costs to the health system [24]. The San Antonio Street Nursing program, which aimed to bring healthcare services directly to unhoused individuals, was organized by the University of Texas Health San Antonio School of Nursing in partnership with San Antonio Street Medicine [25]. Armed with backpacks filled with medical supplies, nursing students rounded the streets of San Antonio to provide basic care, such as wound management and dressing to the unhoused population [25]. Although the Cohealth podiatry service and San Antonio Street Nursing project did not focus on cost analysis, these two programs can be used as models of active outreach to unhoused individuals that can alleviate healthcare costs by treating wounds and infections before they exacerbate into complex, more expensive conditions.

Several studies directly examined the costs and benefits of wound care programs. The JustHealth Recuperative Care Program in Washington State provides unhoused individuals discharged from hospitals a safe place to recover from their injuries with housing, nursing care, wound management, and social services [26]. The total cost per guest per day during this 15-month pilot project was $157.45, which saved acute care facilities between $18,000 and $48,000 per day [26]. The mobile BNEP Wound Clinic reported low costs in treating unhoused individuals with wounds, amounting to $146.65 per client [27]. This is much lower than the per-client cost in clinic-based wound care, which can cost up to almost $5000 [27–29]. The Wound and Abscess Clinic at Casa Segura/Safehouse in Oakland, California, reported a $5 cost per client, excluding overhead [30]. Given that healthcare spending for the unhoused population is nearly three times that for the housed population [31], these programs represent cost-effective wound care models for unhoused individuals.

#### Comprehensive Services

Features such as drop-in service availability [18, 23, 32], HIV or other disease screening [18, 20, 32, 33], syringe exchange [18, 20, 21, 27, 30, 32–34], provision of temporary housing or assistance in securing housing [18, 26, 30, 32, 35], case management [26, 30, 35], preventative education [18, 20], and the incorporation of an interdisciplinary staff [23, 25, 26, 30, 32, 35] contributed to the effectiveness of community-based programs. For example, the Homelessness Team Program at Cohealth in Melbourne, Australia, offered a fixed site and multiple outreach services. In addition to podiatric health services offered at fixed locations to clients, teams provided drop-in medical services in locations where unhoused individuals tend to gather [23]. Drop-in services have proven to be effective for these individuals because they can be reluctant to seek healthcare on their own [16, 23, 36, 37]. Another example is the Dhaka NSEP in Bangladesh, which offered similar drop-in services to its unhoused populations [18]. In an effort to prevent the escalation of an HIV epidemic, this program provided services such as syringe exchange, wound abscess management, rest and recreational facilities, and HIV/AIDS education at seven drop-in centers within the community. Similar to the Cohealth drop-in service in Melbourne, the needle exchange service in Dhaka was conducted via outreach in community areas where injectors were known to gather regularly [18]. The Dhaka NESP also offered HIV, syphilis, hepatitis C, and hepatitis B screenings. Similar services were also offered in IDEA SSP in Miami, Florida [33, 34]. Another program focused on drop-in, and easy-access care was the Community-Based Medication-First (CBMF) Program for opioid use disorder (OUD) in Washington State, which provided same-day medication for individuals suffering from OUD [32].

The provision of temporary housing or assistance in finding housing for clients was another service common to many community-based programs. In four of the community-based healthcare programs already discussed, some type of housing assistance was offered to clients [18, 23, 26, 35]. For example, the main service offered by the JustHealth Recuperative Care Program in Washington State was motel respite care for unhoused individuals following hospital discharge [26]. Respite care services provide a safe environment for unhoused individuals to rest and receive acute medical care and have proven beneficial in reducing the length of hospital stays and decreasing readmission rates [38]. Healthcare for the Homeless in Baltimore, MD, additionally offered respite care services to unhoused individuals. Along with providing medical care, mental health and addiction counseling, chronic disease management, and occupational therapy, this program helped secure temporary and permanent housing for its clients [35].

The incorporation of interdisciplinary care systems also contributed to the success of community-based care programs. Healthcare for the Homeless in Baltimore, the Homelessness Team Program at Cohealth in Melbourne, the JustHealth Recuperative Care Program in Washington State, and others [25, 30] recruited team members from various disciplines, including physicians, nurses, mental health specialists, social workers, case managers, and occupational therapists, to tend to the various needs of their clients. For example, JustHealth case management services included assistance with obtaining valid identification, establishing primary care and legal services, and reconnecting to resources such as Social Security and veteran benefits [26]. Occupational therapy at Healthcare for the Homeless helped unhoused individuals transition into independent housing [35]. Healthcare providers in this program commented on the value of having an occupational therapist’s in-depth understanding of the clients’ day-to-day activities and difficulties to plan their treatment [35].

#### Lessons Learned from Community-Based Programs

The lack of space on the BNEP Wound Clinic mobile recreational vehicles limited the staff to seeing only one client at a time [27]. Further, though many clients had already established rapport with BNEP staff, follow-up care was difficult because many clients did not have access to phones [27]. Since depression was a central factor associated with syringe sharing, the authors from the Tacoma, Washington SEP cross-sectional study recommended that SEPs should incorporate mental health services and health education programs focused on mental health issues [21]. The Wound and Abscess Clinic at Casa Segura/Safehouse in Oakland, California, found that verbal communication, rather than distribution of flyers and other printed materials, was more effective in reaching the unhoused population [30]. Both the IDEA SSP in Miami and Malmo SEP in Sweden found that many of their participants reported wound and skin infections, highlighting the need for wound care clinics at SSP sites [20, 34]. Adding clinics to these SSP sites would allow clients to obtain immediate wound care and promote education on the prevention, recognition, and treatment of wounds [34]. The Dhaka NESP reported a syringe shortage due to fluctuations in outreach activity and resources, which led to risky injection behaviors being relatively common in the sampled group of IDUs [18]. The JustHealth Recuperative Care Program in Seattle, Washington, suggested a better system of tracking clients once they were discharged from the community-based program to allow for a closer follow-up care [26]. The podiatry service at the Homelessness Team Program at Cohealth in Melbourne, Australia, observed that some treatments common in private or facility-based clinics may be impractical at a community-based podiatry service serving the unhoused. Examples included strapping a client’s foot and providing ice and heat packs, since unhoused individuals may not have access to microwaves or freezers [23].

#### Summary of the Literature Review

The narrative literature review found 11 community-based programs that could serve as useful models in the development of a wound care program for unhoused individuals. Important characteristics such as drop-in availability, bloodborne disease screening, temporary housing for clients, preventative education and counseling, and the use of an integrated care team contributed to the effectiveness of these programs in managing health care for marginalized and vulnerable populations. For wound care in particular, clinics that were attached to SEPs were useful in providing clients with immediate, easy-access treatment. Overall, community-based programs were more cost-effective than emergency department visits and acute care facilities and may appeal more to unhoused individuals, due to their easy accessibility, non-judgmental environments, and simpler care system compared to other clinic-based treatments.

### Community-Based Wound Care Services in Hawai'i

#### Description of Clients in Clinic

With an estimated total homeless population of 6458 and 45.6 homeless individuals per 10,000 people, Hawai'i has the second highest population of unhoused individuals per 10,000 people out of all 50 states in the USA, just behind New York, as of 2020 [39]. The community-based wound care program in Honolulu uses the Johns Hopkins Nursing Evidence-Based Practice Model as its framework, which focuses both on building an effective clinical team and on the translation of extensive evidence into individualized treatment for clients [40].

Established in 1993, the CHOW Project’s mission was to promote the optimal health and well-being of people affected by drug abuse. CHOW had five outreach workers, one housing case manager, one research/care coordinator, and three administrative staff: the Executive Director, the Finance Manager, and the Program Manager. CHOW’s social and community health outreach workers collaborated with volunteer nurses, healthcare providers, and students to provide integrated community-based wound care as part of comprehensive harm reduction services to IDUs. CHOW had a mobile van that provided services to participants Monday–Friday at River Street and Vineyard Boulevard on the O’ahu Island location. Since the program was launched, the CHOW Project has merged with another nonprofit, the Life Foundation, and is now known as the Hawaii Health & Harm Reduction Center.

The majority of clients seen were male (66%) with an average age of 43.4 years. The two most self-reported races were White (47%) and Native Hawai'ian (22%). The unhoused condition was reported by 66% of clients, of which 83% had a mental health diagnosis. All clients seen were IDUs. The primary drugs injected included opioids (66%) and methamphetamine (33%). The clients’ self-reported reasons for seeking ED services were primarily detoxification and wound care.

Wound abscesses (26%), skin/soft tissue infections and cellulitis (25%), and venous ulceration (19%) were the most common types of wounds seen and treated. There were about 10 patients referred to The CHOW Project from the Queens Medical Center (QMC), one patient from Castle Medical Center, and over 30 patients referred from the Institute for Human Services. Similarly, about 20 (6%) of CHOW wound care patients were referred to QMC outpatient wound care center, and an estimated 7% were referred to local EDs.

#### Survey Results from Clients

The survey was completed by 46 (84%) of 55 SEP participants, where 39 (85%) clients reported seeking wound care 0–5 times, and 6 (13%) sought care over 20 times in the past 3 months. Forty-four (96%) clients reported needing help keeping wounds clean. Open-ended comments revealed a reluctance to seek treatment at other facilities due to the perception of being “judged” and concerns about long wait times in the ED. Clients also requested wound care supplies and education on how to care for their wounds.

#### Survey Results from Wound Care Providers

Four clinicians, nurses, advanced practice nurses, and physicians from various local organizations, including a local hospital, a federally qualified health center, and a homeless shelter clinic, responded to the provider needs assessment. Most of the clinicians reported that they saw or treated wounds and/or ulcers from clients who sought CHOW services 6–10 times per week, with one response indicating 11–15 times per week. All of the clinicians indicated that the frequency with which they saw wounds related to IDU from CHOW clients was about 0–5 times per week. The most frequently selected types of wounds were related to skin/soft tissue infections and cellulitis, followed by venous, arterial, and traumatic wounds. Half of the healthcare provider respondents indicated that CHOW patients’ access to clean and stable housing was the biggest challenge when caring for a client with IDU-related wounds and/or homelessness, followed by access to wound care supplies. Lastly, 75% of healthcare provider respondents felt that a community-based wound care program would help service the unhoused community and decrease the use of urgent care facilities. All healthcare provider respondents indicated a willingness to collaborate with a community-based wound care program.

#### Cost-Effectiveness of the CHOW Project

Through the seven-month intervention period, $3,491.73 out of $5000 was spent on clinic supplies and necessary resources to operate the community-based wound care program. It is estimated that the average cost to treat a wound care patient was about $33 per patient or about $15 per visit. However, the cost per patient was about $92 when accounting for the estimated cost of hiring a nurse practitioner full-time with benefits. According to the most recent obtainable figures in Hawai'i State in 2012, the average cost per visit to emergency departments for contusion, open wounds, and other trauma to the skin and subcutaneous tissue was $1,613.

#### Lessons Learned from the Community-Based Wound Care Services in Hawai'i

By utilizing a community-based model, CHOW Project clients were able to easily access the wound care services. This was related to the familiarity of the location of the mobile CHOW van, which mainly provided syringe exchange services. Additionally, the existing relationship with CHOW staff fostered a trusting environment for clients to seek services. Providing wound care at the van was compatible with the existing workflow process of the CHOW program despite an increase in workload, given that wound care was newly added to the program.

However, capturing the average time to wound closure as a measured outcome was a challenge. Many clients were lost to follow-up because their wounds improved and they only sought care after a new wound developed, their wound re-opened, or was re-infected. Additionally, another goal at the onset of this project was to decrease inappropriate ED use and overutilization. This was challenging to measure, since unhoused individuals often present to various health systems with acute, immediate health concerns, where they are automatically referred to the ED for more acute-based care.

Another challenge was related to the sustainability of the project, which required ongoing efforts to secure sources of funding and resources. There was a constant need for supplies and equipment to meet evidence-based standards of care. Additionally, access to electronic medical records for patients admitted into the hospital setting presented a challenge. The ability to follow the patient into the inpatient setting would allow the community-based wound care provider to prepare for discharge and better collaborate with the inpatient team. Increasing communication between the hospital and community-based wound care providers may also help decrease the overutilization of ED services and readmissions.

## Discussion

This paper lists published models of community-based programs that can be useful in establishing successful wound care services for unhoused individuals. It also highlights an existing community-based wound care program in Hawai'i to provide critical information on population-specific logistics and policies that must be addressed to meet the medical needs of vulnerable individuals. The open-ended assessment of our clients’ needs and provider experiences shed light on a significant dilemma within the unhoused community with wound care needs, wherein individuals expressed a desire for education about wound care, yet were hesitant to seek treatment due to the social stigmatization associated with their situation. Further, numerous factors associated with the challenges for community-based healthcare programs that we detected through our program in Hawaii have been commonly recognized and encountered in other programs worldwide, as documented in the reviewed literature.

Based on the narrative literature review and the results of the surveys conducted in Hawai'i, the authors identified common factors for successful community-based healthcare programs for unhoused individuals with wounds. First, drop-in services are effective, since the target population is typically reluctant to seek appropriate healthcare services. Second, interdisciplinary teams in community-based clinics can successfully address many of the unique needs of this population, particularly communicable disease screening, syringe exchange, supportive temporary housing, case management, and preventative education [18, 20, 21, 23, 25, 26, 30, 33–35].

Integrating housing services into community-based healthcare programs for unhoused individuals may contribute to the efficiency and efficacy of the provided services, especially for time-intensive services such as wound care. For example, one study reported that the majority of the targeted population needed wound care (91%) and assistance finding stable housing (65%) [26]. Survey responses from healthcare providers in Hawai'i indicated that patient access to clean and stable housing was the biggest challenge in providing a useful community-based healthcare service for IDU-related wounds and/or homelessness. Most of the reviewed articles aligned with findings from the CHOW program of Hawai'i, which revealed the difficulty of follow-up care for unhoused individuals, especially given the lack of access to technologies (such as phones) and the gap in patient knowledge about the need for follow-up care [27]. Providing housing is a crucial means to address these challenges and can significantly contribute to the success of a community-based program.

Important qualities of an effective wound care program include the use of qualified nursing staff, evidence-based patient-centered care, and patient education. One article lists qualities to ensure successful community-based wound care programs [41]. First, recruitment of nursing staff to provide wound care in a community-based clinic showed high levels of wound improvement over time and low wound recurrence [41, 42]. Second, the need for evidence-based practice and consistent, standardized care was also observed to be a critical component for a successful wound care program. Evidence gained through translational research is crucial, as this warrants the implementation of new drugs and devices that can be used in treatment for improved outcomes [41, 43]. However, the translation of research into consistently effective practice is sometimes difficult to achieve [44], since the transient and differing health priorities of unhoused individuals may result in inconsistencies in following up on treatment protocols. For example, diabetic foot ulcers are common among this population and require specialized care [45]. The desired outcome is linked to personal care for the ulcer, which is often hard since unhoused individuals are unable to rest in one spot for an extended period of time [37] or keep an open wound clean enough to heal [37, 45, 46]. Third, active patient engagement during consultations serves as a significant predictor of a successful wound care program. In one study, patients treated for leg ulcers reported that strong confidence in their diagnosis was one of the factors that benefited their treatment most [47], which was a result of a thorough explanation from the care provider. Patients with a complete understanding of their treatment are better suited to implement that treatment after leaving the office. For example, a wound treatment study in Singapore showed that the number of patients who avoided self-wound treatment was mitigated when patients were taught how to properly replace their dressings [48]. Additionally, the Leg Club, a community-based program that treats patients with leg ulcers in the UK, strives to enhance patients’ understanding of their leg condition in order to help them better manage their ulcer care [49]. As a result, patients treated in that program reported decreased levels of pain and increased morale and self-esteem [49].

One very important factor in sustaining community-based programs is the budget. For the Hawai'i CHOW program, the addition of wound care services was a part of a comprehensive harm reduction effort and the cost analysis showed that the cost per patient treated in the CHOW program was much lower than the cost of care provided to patients admitted to the nearby EDs. Thus, cost analysis should be central to community-based health program studies [26, 27, 30]. In addition, the engagement of appropriate stakeholders, private partners, academic institutions, community organizations, and healthcare systems should also be considered in determining overall cost to run these community-based programs. Continued outreach to other community-based organizations and national agencies will also help to ensure sustainability, especially related to supply and financial concerns. Cost analysis will continue to be a primary focus and obtaining more recent cost figures from a statewide perspective can help demonstrate the utility of these community-based programs. One limitation of the study was in the administration of the survey. Only clients in one CHOW site were surveyed, restricting the findings to a specific population or infrastructure, and hence may not be applicable to other locations or programs.

In conclusion, this paper provides a concrete list of community-based programs that address the wound care needs of unhoused individuals and provides an exemplar of a community-based program that addresses the wound care needs of unhoused individuals. Findings from this report may help other communities initiate a high-quality, effective, and sustainable community-based healthcare program for unhoused populations with wounds. Furthermore, as a future direction, we acknowledge the imperative for a more extensive dataset comprising successful instances of community-based healthcare programs to foster the understanding and establishment of such initiatives. Consequently, an interventional study, predicated upon the challenges elucidated by the present findings, should be conducted, with the overarching objective of formulating standardized care protocols for unhoused individuals afflicted with wounds.

## Data Availability

Requesting data from literature review and surveys mentioned in this paper can be made by contacting the authors of this paper.

## Abbreviations

US: The United States
ED: Emergency department
CHOW: The Community Health Outreach Work
IDU: Intravenous drug users
SEP: Syringe exchange program
BNEP: The Baltimore Needle Exchange Program
NSEP: Needle-syringe exchange program
IDEA: The Infectious Disease Elimination Act
SSP: Syringe services program
HIV: Human immunodeficiency virus
CBMF: The Community-Based Medication-First
OUD: Opioid use disorder
QMC: Queen’s Medical Center
